# Hydrogel-Forming Microneedles Prepared from “Super Swelling” Polymers Combined with Lyophilised Wafers for Transdermal Drug Delivery

**DOI:** 10.1371/journal.pone.0111547

**Published:** 2014-10-31

**Authors:** Ryan F. Donnelly, Maelíosa T. C. McCrudden, Ahlam Zaid Alkilani, Eneko Larrañeta, Emma McAlister, Aaron J. Courtenay, Mary-Carmel Kearney, Thakur Raghu Raj Singh, Helen O. McCarthy, Victoria L. Kett, Ester Caffarel-Salvador, Sharifa Al-Zahrani, A. David Woolfson

**Affiliations:** 1 School of Pharmacy, Queen's University Belfast, Belfast, Co. Antrim, United Kingdom; 2 School of Pharmacy, Zarqa University, Zarqa, Jordan; Institute for Frontier Medical Sciences, Kyoto University, Japan

## Abstract

We describe, for the first time, hydrogel-forming microneedle arrays prepared from “super swelling” polymeric compositions. We produced a microneedle formulation with enhanced swelling capabilities from aqueous blends containing 20% w/w Gantrez S-97, 7.5% w/w PEG 10,000 and 3% w/w Na_2_CO_3_ and utilised a drug reservoir of a lyophilised wafer-like design. These microneedle-lyophilised wafer compositions were robust and effectively penetrated skin, swelling extensively, but being removed intact. In *in vitro* delivery experiments across excised neonatal porcine skin, approximately 44 mg of the model high dose small molecule drug ibuprofen sodium was delivered in 24 h, equating to 37% of the loading in the lyophilised reservoir. The super swelling microneedles delivered approximately 1.24 mg of the model protein ovalbumin over 24 h, equivalent to a delivery efficiency of approximately 49%. The integrated microneedle-lyophilised wafer delivery system produced a progressive increase in plasma concentrations of ibuprofen sodium in rats over 6 h, with a maximal concentration of approximately 179 µg/ml achieved in this time. The plasma concentration had fallen to 71±6.7 µg/ml by 24 h. Ovalbumin levels peaked in rat plasma after only 1 hour at 42.36±17.01 ng/ml. Ovalbumin plasma levels then remained almost constant up to 6 h, dropping somewhat at 24 h, when 23.61±4.84 ng/ml was detected. This work represents a significant advancement on conventional microneedle systems, which are presently only suitable for bolus delivery of very potent drugs and vaccines. Once fully developed, such technology may greatly expand the range of drugs that can be delivered transdermally, to the benefit of patients and industry. Accordingly, we are currently progressing towards clinical evaluations with a range of candidate molecules.

## Introduction

Microneedle (MN) arrays, micron scale, minimally-invasive devices that painlessly by-pass the skin's *stratum corneum* barrier, have been shown to be extremely effective in enhancing transdermal delivery of water soluble drugs, biomolecular therapeutics and vaccines [1]–[3]. The compounds delivered to date have typically been of high potency, meaning only a low dose is required to achieve a therapeutic affect (e.g. insulin) [4], [5] or elicit the required immune response [6], [7]. Clearly, the majority of marketed drug substances, including many antibodies, are not low dose, high potency molecules. Indeed, many drugs require doses of several hundred milligrams per day in order to achieve therapeutic plasma concentrations in man. Until now, such high doses could not be delivered transdermally from a patch of reasonable size, even for molecules whose physicochemical properties are ideal for passive diffusion across the skin's *stratum corneum* barrier. Therefore, transdermal delivery has traditionally been limited to fairly lipophilic, low molecular weight, high potency drug substances. Since most drugs do not possess these properties, the transdermal delivery market has not expanded beyond around 20 drugs. Marketed MN-based patches are likely to increase this number of drugs in the coming years. However, this increase will only be maximised if high dose molecules can be delivered in therapeutic doses using MN. We have previously shown that suitably-formulated dissolving MN platforms can deliver therapeutic doses of a low potency, high dose drug substance [8]. However, deposition of polymer in skin from a dissolving MN system may be undesirable if the system is to be used on an ongoing basis. The dissolving MN system employed in this previous study would deposit approximately 5–10 mg of polymer per cm^2^ in skin [8]. If the patch size were 10 cm^2^, then 50–100 mg of polymer would be deposited in the patient's skin every time the product is applied. While vaccines are used infrequently, most therapeutic agents need to be administered regularly. Accordingly, dissolving MN systems may be most appropriate to rapid delivery of low dose vaccines [6], [9].

**Figure 1:**
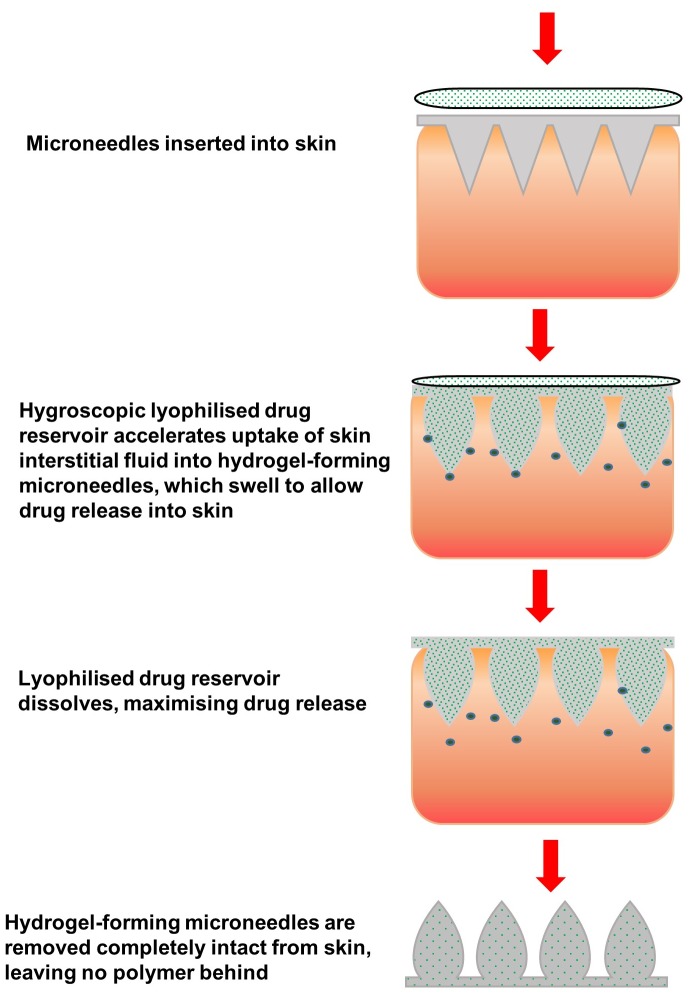
Schematic representation of the concept of combining hydrogel-forming microneedles prepared from super swelling polymers and lyophilised wafer-type drug reservoirs for enhanced transdermal delivery of proteins and high dose low potency drug substances.

We have recently described novel hydrogel-forming MN arrays, prepared under ambient conditions that contain no drug themselves [10]. Instead, they rapidly imbibe skin interstitial fluid upon insertion to form continuous, unblockable conduits between the dermal microcirculation and an attached patch-type drug reservoir. Such hydrogel-forming MN initially act simply as a tool to pierce the *stratum corneum* barrier. Upon insertion, they function as a rate-controlling membrane, allowing sustained drug delivery at a rate controllable by adjustment of crosslink density, which dictates swelling rate [10], [11]. Importantly, such MN are removed intact from skin, leaving no measurable polymer residue behind, but are sufficiently softened, even after 1 minute of skin insertion to preclude reinsertion, thus further reducing the risk of transmission of infection [12]. In the present study, we substantially modified this novel system to facilitate delivery of clinically-relevant doses of a low potency, high dose drug substance and rapid delivery of a model protein by using a modifying agent to increase swelling capabilities [13] and using a hygroscopic lyophilised drug reservoir (**Figure 1**).

## Methods and Materials

### 2.1. Chemicals

Polyethylene glycol (PEG, MW 10,000 Da), ibuprofen-sodium, chicken ovalbumin (OVA, albumin from chicken egg, grade IIV), mouse monoclonal anti-chicken ovalbumin antibody (moAb), Ph. Eur. Gelatin, D-mannitol and tetramethylbenzidine (TMB) substrate were purchased from Sigma-Aldrich, Dorset, UK. Phosphate buffered saline tablets, pH 7.4 (PBS) were obtained from Oxoid Ltd., Hampshire, UK. Rabbit anti-ovalbumin horse-radish peroxidase (HRP) conjugated-polyclonal antibody was purchased from Gene Tex Inc Alton Pkwy, Irvine, USA. SuperBlock T20 was purchased from Thermo Scientific, Rockford, Illinois, USA. 50C-Mannitol was supplied by Roquette, Lestrem, France. Gantrez AN-139 and S-97, copolymers of methyl vinyl ether and maleic anhydride and methyl vinyl ether and maleic acid, respectively (PMVE/MAH and PMVE/MA, with molecular masses of 1,080,000 and 1,500,000 respectively) were gifts from Ashland, Kidderminster, UK. Unless otherwise stated, all other chemicals and materials were supplied by Sigma-Aldrich (Dorset, UK) or Fisher Scientific (Loughborough, UK).

### 2.2. Preparation of hydrogel films

The aim here was to investigate various polymeric compositions in order to find a material capable of rapid swelling, but which would be sufficiently hard in the dry state to penetrate the skin. Importantly, once swollen, the material should maintain structural integrity and be reasonably robust during handling.

**Figure 2:**
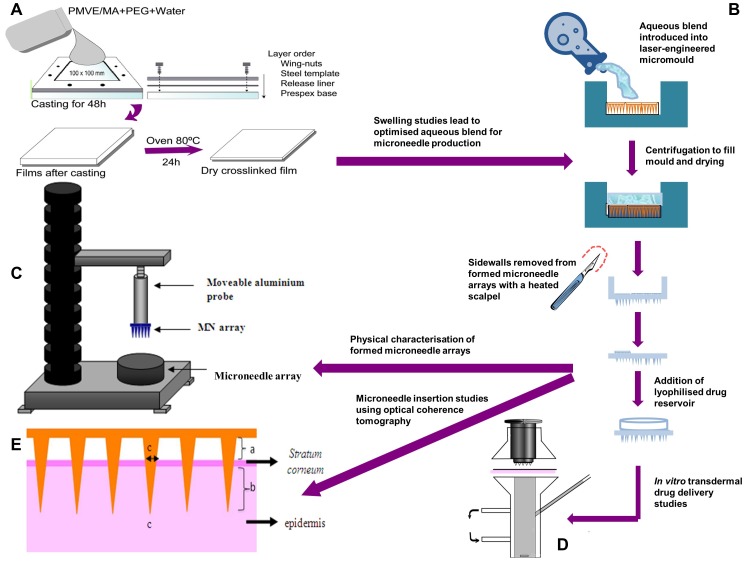
Schematic representation of casting and crosslinking of the super swelling hydrogel films (A), microneedle preparation (B), Texture Analyser set-up for investigation of physical properties of microneedles (C) and Franz cell set-up for *in vitro* transdermal drug release studies (D). Panel (**E**) shows a diagrammatic representation of the measurements recorded from the optical coherence tomographic images of microneedle penetration into excised neonatal porcine skin *in vitro*, namely; (a) the distance between the lower microneedle base plate and the *stratum corneum*, (b) the depth of microneedle penetration into the skin and (c) the width of the micropore created in the skin.

Stock solutions of Gantrez S-97 (40% w/w) or AN-139 (30% w/w) [13], [14] were prepared using deionised water (**Figure 2A**). Hydrogel films were then prepared using varying concentrations of the co-polymer, PEG 10,000 and the modifying agent, sodium carbonate (Na_2_CO_3_). The blends were centrifuged at 3,500 rpm for 15 minutes in order to remove any air bubbles. The aqueous blends (30 g) were then slowly poured into moulds consisting of a release liner with the siliconised surface facing upwards (Rayven, Inc., Saint Paul, MN, USA) secured to a Perspex base plate with stainless steel clamps. Once assembled, the internal dimensions available for casting were 100 mm×100 mm. The aqueous blends were spread evenly across the moulds and these were placed onto a level surface. The cast blend was dried for 48 hours at room temperature. After drying, the films were cured at 80°C for 24 h to induce chemical crosslinking between the PMVE/MA and PEG by ester formation [14], [15]. Films were then removed from the mould by simply peeling the release liner, with attached film, off the base.

### 2.3. Swelling of hydrogel films in phosphate-buffered saline (PBS)

For swelling studies, individual film portions (1 cm^2^) were weighed at the zero time point in the dry state (m_0_) and then placed into a volume of PBS (pH 7.4). The film portions were removed at specific time points, surface fluid was removed between pieces of filter paper and the mass of the swollen film was recorded (m_t_). PBS was selected as the swelling medium, as it was deemed to closely resemble/simulate skin interstitial fluid and has been used as the swelling medium in other similar studies [14]. The percentage swelling of the film was determined using **Equation 1**.
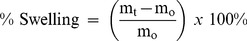
(1)To examine the controlled swelling mechanism of the PEG-crosslinked PMVE/MA hydrogels, a second order kinetic model was used to process the experimental data, as outlined in **Equation 2**, where A is the reciprocal of the initial swelling rate of the hydrogel, *r_o_*, or 1/(*k*_s_ *S*_eq_^2^), where *k_s_* is the swelling rate constant and B is the inverse of the degree of swelling at equilibrium, *S*_eq_ [16].
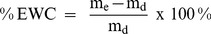
(2)To analyse the kinetic model, *t/S* versus *t* graphs were plotted and respective swelling rate parameters were determined [14]. The dynamics of the water sorption process are usually investigated either by monitoring the change in physical dimensions of the swelling hydrogel or by knowing the amounts of water imbibed by the hydrogel at various time points. In the current study, the latter procedure was engaged. Analysis of the swelling kinetics of the various films was carried out using **Equation 3** [14]. The portion of the water absorption curve with a fractional water uptake (*M_t_/M_∞_*) less than 0.60 was analyzed with **Equation 4**, where *M_t_* is the mass of water absorbed at time t, *M_∞_* is the water uptake at equilibrium. *k* is a gel characteristic constant, which depends on the structural characteristics of the polymer and its interaction with the solvent and *n* is the swelling exponent, describing the mechanism of penetrant transport into the hydrogel. The constants *n* and *k* may be calculated from the slopes and intercepts of the plots of ln(*M_t_/M_∞_*) versus ln *t* from the experimental data. The value of n provides an indication of the water transport mechanism. When n = 0.5, the swelling process is of Fickian nature and is diffusion controlled while the value of n between 0.5 and 1 suggests non-Fickian diffusion or more specifically anomalous diffusion. When n becomes exactly equal to unity, then the diffusion is termed case II diffusion. In some cases, the value for n has been found to exceed unity and this represents super case II transport [17]–[19].
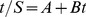
(3)

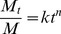
(4)Hydrogel network structure characterization is a complex procedure because of the many types of possible networks, including, regular, irregular, loosely/highly cross-linked and imperfect networks. As a result of these variations in the network structure, only average values for the cross-linking density and molecular weight (MW) between crosslinks are represented using different experimental or theoretical methods [13], [16]–[19]. In the present study, the number average MW between cross-links, 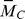, was determined using equilibrium swelling theory, 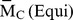, rather than glass transition temperature. The magnitude of 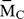 affected the mechanical and physical properties of crosslinked polymers. The volume fraction of a polymer, 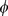, in the swollen state describes the amount of liquid that can be imbibed into a hydrogel and is described as a ratio of the polymer volume to the swollen gel volume (**Equation 5** **and** **6**).
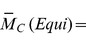
(5)

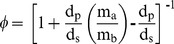
(6)Here, V_s_ is the molar volume of water (18 cm^3^/mol), 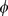 is volume fraction of polymer in the hydrogel, χ is the Flory-Huggins polymer-solvent interaction parameter; In the above Equation, m_a_ and m_b_ are the mass of polymer before and after swelling and d_ρ_ and d_s_ are the densities of polymer and solvent, respectively. The density of the polymeric films was calculated using the following formula; d_ρ_ *= w/SX*, where; *X* is the average thickness of the film, S is the cross-sectional area and *w* weight of the film. The polymer water interaction parameter (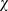) reflects the thermodynamic interaction in hydrogels, which in turn indicates the change of interaction energy when polymer and solvent mix together. The 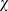 parameters of hydrogels can be obtained experimentally *via* **Equation 7** [13], [16]–[19].
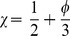
(7)
**Equation 7** neglects the *M_c_* dependence of the *χ* parameter, and therefore, this equation indicates that the *χ* values are always ≥0.50. In the present study, crosslink density, *V_e_*, was determined using **Equation 8**. *V_e_* represents the number of elastically effective chains, totally induced in a perfect network, per unit volume. Where, N_A_ is Avagadro's number (6.023×10^23^ mole^−1^) [13], [16]–[19].
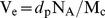
(8)


### 2.4. Fabrication of hydrogel forming MN arrays

Formulations used to prepare MN were based upon the preceding swelling studies. Aqueous blends containing 15% w/w Gantrez AN-139 and 7.5% w/w PEG, 10,000 (control formulation) [14] were utilized to fabricate MN arrays as previously described [10], [11]. The blend (500 mg) was poured into MN moulds (361 (19×19) needles perpendicular to the base and of conical shape, 600 µm high with base width of 300 µm and 50 µm interspacing on a 0.49 cm^2^ patch) and these were centrifuged at 3,500 rpm for 15 min and dried at room temperature for 48 h. MN were crosslinked (esterification reaction) by heating at 80°C for 24 hours [10], [11], [14] and the sidewalls formed by the moulding process were removed using a heated blade (**Figure 2B**). MN arrays were also prepared from a so-called “super swelling” hydrogel formulation containing 20% w/w Gantrez S-97, 7.5% w/w PEG 10,000 and 3% w/w Na_2_CO_3_.

### 2.5. Mechanical characterisation of super swelling microneedle arrays

MN were subjected to standard mechanical tests using a TA-XT2 Texture Analyser (Stable Microsystems, Haslemere, UK) in compression mode, as described previously [10], [11], [20]. Briefly, MN arrays were visualised before testing using a light microscope (GXMGE-5 digital microscope, Laboratory Analysis Ltd, Devon, UK). MN arrays were then carefully placed on the flat stainless steel baseplate of the Texture Analyser with the needles pointing upwards. A flat-faced probe with a diameter of 11.0 mm was lowered at a speed of 0.5 mm s^−1^. Upon contact with the MN array, the probe continued to travel at a speed of 0.5 mm s^−1^ until the required force had been exerted. Once the target force was reached, the probe was moved upwards at a speed of 0.5 mm s^−1^. MN arrays were then viewed again under the light microscope.

### 2.6. Skin insertion studies

Neonatal porcine skin, a good model for human skin [21], [22], was obtained from stillborn piglets and immediately (<24 hours after birth) excised, trimmed to a thickness of 700 µm using a dermatome (Integra Life Sciences, Padgett Instruments, NJ, USA) and frozen in liquid nitrogen vapour, as previously described [10], [11], [20]. Skin was then stored in aluminium foil at −20°C for no more than 7 days prior to use.

Skin was mounted on the baseplate of the Texture Analyser using cyanoacrylate adhesive (Loctite Ltd, Dublin, Ireland) while the MN were this time attached to the probe using double-sided tape (3M, Carrickmines, Ireland). The probe then moved downwards as described above until the required force had been exerted. Once the target force was reached, the probe was moved upwards at a speed of 0.5 mm s^−1^. The number of MN in an array that had penetrated the skin's *stratum corneum* barrier was counted following visualisation of the pores formed in skin using methylene blue solution (1 mg/ml in PBS pH 7.4).

Optical coherence tomography (OCT), as described previously [23] allowed measurement of the depth of MN insertion for each application force, since the MN are transparent and accordingly can be left in place during OCT studies to mimic their intended use (EX1301 OCT microscope, Michelson Diagnostics, Kent, UK). OCT was also used to visualise the *in situ* swelling of the MN in real time skin at varying time intervals over a 3 h period. OCT data files were exported to Image J (National Institutes of Health, Bethesda, MD, USA) for measurement of insertion depth and false colours were applied using Ability Photopaint (Ability Software International, Horley, UK) for presentation purposes.

### 2.7. Preparation and characterisation of lyophilised drug reservoirs

A range of lyophilised wafer-type reservoirs loaded with the model compounds ovalbumin (OVA) or ibuprofen sodium were prepared containing varying concentrations of gelatin, mannitol and, in some instances, sodium chloride (NaCl) and sucrose. In the case of the ibuprofen sodium wafers, a variety of different gelatin sources, all at loadings of 10% w/w, were tested in combination with 3% w/w mannitol and 40% w/w ibuprofen sodium in deionised water.

To prepare OVA-containing reservoirs, the protein (0.5% w/w) was dissolved in distilled water, followed by the addition of gelatin, mannitol, NaCl and sucrose. It was then mixed by speed mixer at 3,000 rpm for 60 s and sonicated at 37°C for 60 min. To prepare the ibuprofen sodium-loaded reservoirs, the individual components were mixed by speed mixer at 3,000 rpm for 60 s and sonicated at 37°C for 60 min. The resulting OVA or ibuprofen-sodium formulations were then cast (500 mg in the case of OVA-loaded wafers and 250 mg in the case of ibuprofen sodium-loaded wafers) into cylindrical moulds with one open end moulds (diameter 15 mm, depth 5 mm), frozen at −80°C for a minimum of 60 min and then lyophilised in the freeze-drier (Virtis Advantage Bench top Freeze Drier System, SP Scientific, Warminster PA, USA), according to the following regime: primary drying for forty eight hours at a shelf temperature of −40°C, secondary drying for ten hours at a shelf temperature of 20°C and vacuum pressure of 50 mTorr.

Dried reservoirs were characterised using standard pharmacopoeial tests. Twenty reservoirs were selected randomly, weighed individually and their average weight was calculated to determine the weight uniformity. The percentage deviation of each reservoir from the average weight was determined. The thickness of the reservoirs was determined with a digital micrometer (Digital Calliper, 0–150 mm, Jade Products Rugby Limited, Warwickshire, UK). Five reservoirs were used and average values were calculated. Hardness was determined using a Copley Hardness Tester (Copley Scientific, Nottingham, UK). Twenty reservoirs were weighed (W_0_) and then placed in the Friabilitor (Copley Scientific, Nottingham, UK). This was operated at 25 rpm for 4 min. The reservoirs were then weighed again (W). The % friability was then calculated using **Equation 9**. OVA and ibuprofen sodium contents were determined using the ELISA and HPLC methods described below following dissolution of the reservoirs in PBS pH 7.4.
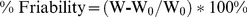
(9)


### 2.8. *In vitro* release studies

The diffusion of ibuprofen sodium (MW 228.26 g/mol) and OVA (MW 45,000 g/mol) from lyophilised active-loaded tablets through hydrogel MN arrays and across neonatal porcine skin was investigated *in vitro* using modified Franz diffusion cells (FDC-400 flat flange, 15 mm orifice diameter, mounted on an FDCD diffusion drive console providing synchronous stirring at 600 rpm and thermostated at 37±1°C, Crown Glass Co. Inc., Sommerville, NJ, USA), as described previously [4]. Briefly, neonatal porcine skin was obtained from stillborn piglets and immediately (<24 hours after birth) excised and trimmed to a thickness of 350 µm using an electric dermatome. Skin was then stored in aluminium foil at −20°C until required. Skin barrier function integrity was confirmed in all cases using standard transepidermal water loss measurements (VapoMeter, Delfin Technologies Ltd, Kuopio, Finland), with any damaged skin immediately discarded. Neonatal porcine skin samples were then shaved carefully so as not to damage the skin (again confirmed by TEWL) and were then pre-equilibrated in phosphate buffered saline (PBS), pH 7.4, for 15 minutes prior to the commencement of experimentation. A circular specimen of the skin was secured to the donor compartment of the diffusion cell using cynoacrylate glue (Loctite, Dublin, Ireland) with the *stratum corneum* facing towards the donor compartment. This was then placed on top of dental wax, to give the skin support, and MN arrays inserted into the centre of the skin section, using a spring activated applicator at a force of 11 N/array. The lyophilised active-loaded wafers (which had been trimmed to size using a heated scalpel blade) were placed on the top of the MN, with 20 µl water used to initiate adhesion. A tubular stainless steel weight (diameter 11.0 mm, 3.5 g mass) was then placed on top of this. The donor compartments were mounted onto the receptor compartments and the Franz cell donor compartments covered with laboratory film (Parafilm, Pechiney Plastic Packaging, WI, USA) so as to avoid evaporation of PBS over the course of experimentation. At predetermined time intervals, a 200 µl sample was collected *via* the side arm of the Franz cell and the receiver compartment immediately replenished with an equivalent volume of release medium. OVA was quantified again using ELISA, while ibuprofen sodium was determined using HPLC.

### 2.9. *In vivo* evaluation of OVA and ibuprofen sodium delivery through super swelling MN

Prior to experimentation, rats were acclimatised to laboratory conditions for a 7 day period. Super swelling hydrogel MN arrays were manually inserted into the skin at a site on the rats' backs. An aliquot of water (20 µl) was applied to the centre of the array and the lyophilised reservoir (again trimmed to size) was placed on top of this. A bespoke adhesive film (Scotchpak 9732, 3M, Carrickmines, Ireland, coated with a 1.0 mm layer of DuroTak 34-416A, National Starch & Chemical Company, Bridgewater, NJ, USA) was applied on top of, and around the edges of, the lyophilised reservoirs to aid retention and provide occlusion. Following application of this integrated system, blood samples were collected at pre-defined time points over 24 h for analysis.

All animal experiments throughout this study were conducted according to the policy of the federation of European Laboratory Animal Science Associations and the European Convention for the protection of vertebrate animals used for experimental and other scientific purposes, with implementation of the principles of the 3R's (replacement, reduction, refinement). Ethical permission specifically for the experiments described here was obtained from the Queen's University Animal Welfare and Ethics Review Board and all researchers carrying out the work had Personal Licences from the UK Home Office. To anaesthetise the animals, isoflurane was used and carbon dioxide was used for euthanisation.

### 2.10. Extraction of plasma and drug

The following procedure was carried out in the case of ibuprofen sodium-containing samples only. Control rat blood for method development was obtained from healthy Sprague dawley rats. Blood from culled rats was collected via heart puncture with a heparinised syringe into ethylenediaminetetraacetic acid (EDTA)-coated tubes. Plasma separation was performed by centrifuging the blood at 500× g for 10 min in a refrigerated centrifuge (4°C). The plasma was then aliquoted into microtubes and stored at −80°C until required. In the case of standards used in assay development, 10 µl of ibuprofen-sodium working standard solutions were added to 190 µl blank plasma. In the case of plasma samples from MN-treated rats, the drug was extracted from the samples without the addition of any endogenous drug. Samples were then vortex mixed for 10 s in a poly(propylene) microtube and 500 µl each acetonitrile (ACN) was added. The samples were vortex mixed for 10 min and centrifuged at 14,000× g for 10 min at 4°C. The ACN extraction procedure was then repeated to ensure optimum extraction of the drug. The sample mixture was placed in a disposable glass culture tube and the extract dried under a stream of nitrogen at 35°C for 50 min using a Zymark TurboVap LV Evaporator Workstation (McKinley Scientific, Sparta, NJ, USA). The residue was then reconstituted in 200 µl PBS (pH 7.4) and collected into a microtube. This was then vortex mixed for 30 s and centrifuged at 14,000× g for 10 min at room temperature. The supernatant was transferred into an auto sampler vial and 50 µl was injected onto the HPLC column and detection carried out as outlined below. In the case of blood samples collected from OVA- treated rats, plasma was separated from whole blood as outlined and this was then quantified by ELISA experiments.

### 2.11. Pharmaceutical analysis of ibuprofen sodium

Ibuprofen sodium quantification in PBS (pH 7.4) and rat plasma was performed using reverse-phase high performance liquid chromatography (RP-HPLC) (Agilent 1200 Binary Pump, Agilent 1200, Standard Autosampler, Agilent 1200 Variable Wavelength Detector, Agilent Technologies UK Ltd., Stockport, UK) with UV detection at 220 nm. Gradient separation was achieved using an Agilent Eclipse XDB-C18 (5 µm pore size, 4.6×150 mm) analytical column fitted with a guard cartridge of matching chemistry. The mobile phase was 60%∶40% methanol∶10 mM potassium phosphate (pH 4.6), with a flow rate of 1 ml min^−1^, and a run time of 30 min per sample. The injection volume was 50 µl. The chromatograms obtained were analysed using Agilent ChemStation Software B.02.01. Least squares linear regression analysis and correlation analysis were performed on the calibration curves produced, enabling determination of the equation of the line, its coefficient of determination and the residual sum of squares (RSS). To determine the limit of detection (LoD) and limit of quantification (LoQ), an approach based on the standard deviation of the response and the slope of the representative calibration curve was employed, as described in the guidelines from the International Conference on Harmonisation (ICH) [24]. Ibuprofen sodium, either dissolved in PBS (pH 7.4) (standards), or samples collected from the Franz cell apparatus (unknowns), was quantified by injection of the sample, following filter sterilisation through 0.2 µm filters, directly onto the HPLC column. In the case of plasma samples, the drug was first extracted from the plasma as described above and then the resulting sample, which had been reconstituted in PBS (pH 7.4) and filtered through a 0.2 µm filter, was injected onto the column. The method parameters for detection of ibuprofen sodium in PBS (pH 7.4) and plasma were identical.

### 2.12. Enzyme-linked immunosorbent assay (ELISA) for detection of OVA

An OVA ELISA, developed as described previously [25], was used to detect OVA in samples collected in both *in vitro* and *in vivo* experiments. Briefly, monoclonal anti-chicken egg albumin (ovalbumin) antibody produced in mouse (moAb) was diluted in 0.1 M bicarbonate buffer, pH 9.6 to the optimized concentration of 2.5 µg/ml. An aliquot (50 µl) of anti-ovalbumin was dispensed into the plate and incubated overnight at 4°C. The plate was filled with washing buffer 0.05% v/v Tween-PBS and soaked for 30 seconds before discarded. This process was repeated 5 times. Then, the plate was turned onto absorbance paper to remove any remaining buffer. The plate was blocked with SuperBlock T20 buffer (150 µl/well) and incubated for 2 hours at room temperature. For the calibration curve, OVA solutions were freshly prepared at a concentration of 1 mg/ml in PBS produced concentrations of 1 µg/ml to 10 ng/ml. A 50 µl of sample was dispensed into wells, each sample was analysed in triplicate. The plate was covered and incubated for 1 hour at room temperature. The plate was washed and incubated with rabbit anti chicken OVA polyclonal antibody conjugate with horse-radish peroxidase (HRP) at the optimized concentration of 5 µg/ml in SuperBlock T20 buffer for 1 hour at room temperature. After the plate was washed, 50 µl of TMP was added to each well to detect antibody binding and incubated for 15 min. Colour development was ended using 50 µl/well of 4.0 M HCl and optical density was measured at 450 nm using a microplate reader spectrophotometer (EnSpire Multimode Plate Reader, PerkinElmer, Waltham, MA, USA). In terms of the analysis of blood samples collected during *in vivo* experiments, plasma was separated from whole blood as outlined below and this plasma was then subjected to the ELISA protocol outlined above.

### 2.13. Statistical analysis

Data was analysed, where appropriate, using the Student's t-test, one-way analysis of variance ANOVA, Mann-Whitney U-test or Wilcoxon test. In each case, a *p*-value less than 0.05 was considered to denote significance.

## Results and Discussion

Microneedles (MN) prepared from hydrogel-forming materials have a range of advantageous characteristics. Firstly, the delivered dose is not limited by what can be loaded into, or onto the surface of the needles themselves, since the drug is contained within an attached patch-type drug reservoir. Secondly, controlled administration is possible for the first time with MN. Finally, since the MN are removed intact, no polymer is deposited in skin, while the inherent antimicrobial properties of the polymer composition used and the fact that the needles are soft upon removal mean transmission of infection from patient to patient is unlikely. To date, we have employed such systems in the effective *in vivo* delivery of potent biomolecules, such as insulin, and for sustained administration of proteins and small molecules over hours or days [10]–[12]. In order to take this technology to the next stage of development, we must now move beyond simply controlling transdermal permeation to demonstrate its utility in administration of clinically-relevant doses of non-potent drugs and in rapid delivery of large molecules with physicochemical properties similar to vaccine antigens. Accordingly, in the present study, we used our previous experience with hydrogels [13] to alter the MN formulation to enhance its swelling capabilities and changed the drug reservoir from a flexible polymeric patch to a lyophilised wafer-like design. Using a hygroscopic reservoir is likely to have two principal effects. Firstly, its high solid content and porous nature will attract water from skin interstitial fluid by osmosis through the hydrogel-forming MN, whose swelling rate will be enhanced. Secondly, since such lyophilised systems are rapidly soluble in water, the rate of drug dissolution and its subsequent availability for diffusion will also be increased. These theories were examined for the first time here using ibuprofen sodium and ovalbumin as model compounds. Importantly, we also examined MN arrays post removal, since it was possible that excessive swelling would reduce mechanical integrity.

### 3.1. Swelling studies

**Figure 3:**
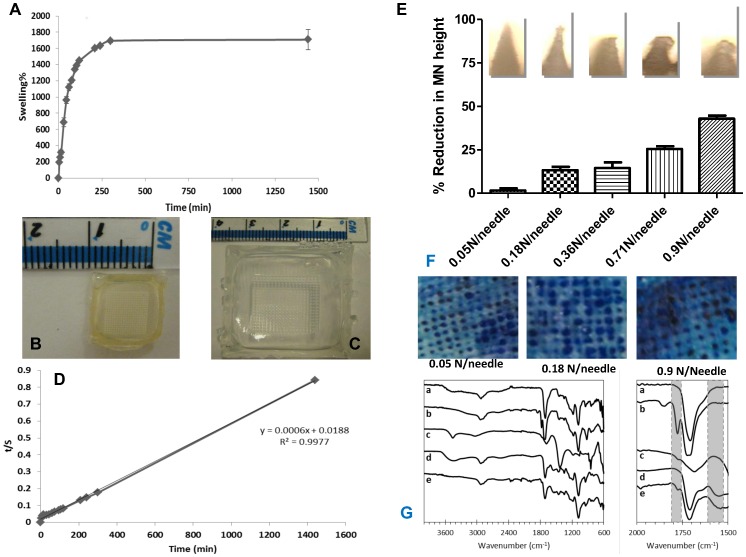
Swelling curve for crosslinked hydrogel films prepared from aqueous blends containing 20% w/w PMVE/MA, 7.5% w/w PEG and 3% Na_2_CO_3_ based on the increasing mass of the swelling array expressed as a percentage of the mass of a dry array (Means ± SD, n = 3) (A). Super swelling microneedle arrays prepared from aqueous blends containing 20% w/w PMVE/MA, 7.5% w/w PEG and 3% Na_2_CO_3_ as (**B**) xerogel and (**C**) post-swelling for 3 hours in PBS pH 7.4. t/S versus t swelling curves of super swelling hydrogel prepared from aqueous blends containing 20% w/w PMVE/MA, 7.5% w/w PEG and 3% Na_2_CO_3_ (Mean ± SD, n = 3) (**D**). Digital microscope images of super swelling hydrogel-forming MN (prepared from aqueous blends containing 20% w/w PMVE/MA, 7.5% PEG 10,000 and 3% Na_2_CO_3_) following the application of different forces (0.05, 0.18, 0.36, 0.71 and 0.9 N/needle). These images are representative of the percentage reduction in the heights of needles on the MN arrays observed following the application of the different forces (Means+SD, n = 3) (**E**). Digital images showing micropores in excised neonatal porcine skin following application of different forces and subsequent staining with methylene blue solution post microneedle removal (**F**). Attenuated total reflectance (ATR)-Fourier transform infrared (FTIR) spectra of dry hydrogels prepared from aqueous blends containing: 20% w/w Gantrez S-97, 7.5% w/w PEG 10.000 non crosslinked (a) and crosslinked (b) materials; Na_2_CO_3_ (c) and 20% w/w Gantrez S-97, 7.5% w/w PEG 10.000 and 3% w/w Na_2_CO_3_ non crosslinked (d) and crosslinked (e) materials. The left panel shows a closer view of the carbonyl region for the same materials. A FTIR Accutrac FT/IR-4100 Series (Jasco, Essex, UK) equipped with MIRacle diamond ATR was used at room temperature. Samples were scanned and recorded in the region of 4000–400 cm^−1^ at a resolution of 4.0 cm^−1^. The obtained spectra were an average of 64 scans. A standard smoothing process was applied to all the spectra using the equipment software (**G**).

The results outlined in **Figure 3A** display the percentage swelling in PBS pH 7.4 of super swelling hydrogel films cast from aqueous blends of 20% w/w Gantrez S-97, 7.5% w/w PEG 10,000 and 3% w/w Na_2_CO_3_. The hydrogel formulation incorporating Na_2_CO_3_ as a modifying agent showed greater initial swelling and reached equilibrium more quickly than the control formula (15% Gantrez AN-139, 7.5% PEG). For example, after 1 hour, the percentage swelling of super swelling hydrogels (20% w/w Gantrez S-97, 7.5% w/w PEG and 3% w/w Na_2_CO_3_) was 1119%, compared to only 250% for the control formulation. Hydrogels prepared from aqueous blends containing 3% w/w Na_2_CO_3_ showed a significant (*p*<0.05) increase in percentage swelling. The percentage swelling at equilibrium was 1071% and 1708% for the control and super swelling formulations, respectively (**Table 1**). **Figure 3** also illustrates the morphology of the MN array as a xerogel (B) and post-swelling in PBS (C). **Figure 3D** is representative of the liner regression plots derived from swelling curves using Equation 3.

**Table 1:** Aqueous blends used to prepare hydrogel formulations tested in the current study and the equilibrium swelling of the formed hydrogels (Means ± SD, n = 3).

Formulation no.	Ingredients (pH value of Gantrez gel prior to addition of other ingredients and making up to volume with deionised water)	Percentage swelling at equilibrium
Control formulation	15% w/w Gantrez AN-139 (pH 2) 7.5% w/w PEG 10,000	1071±106
1	15% w/w Gantrez S-97, (pH 2) 7.5% w/w PEG 10,000	918±13
2	20% w/w Gantrez S-97, (pH 2) 7.5% w/w PEG 10,000, 3% w/w Na_2_CO_3_	1708±125
3	15% w/w Gantrez AN-139, (adjusted to pH 4) 7.5% w/w PEG 10,000, 3% w/w Na_2_CO_3_	Dissolved
4	16% w/w Gantrez AN-139, (adjusted to pH 4) 6% w/w PEG 10,000, 3% w/w Na_2_CO_3_	Dissolved

Using attenuated total reflectance (ATR)-Fourier transform infrared (FTIR) spectroscopy, the mechanism of action of the modifying agent was confirmed to be due to sodium salt formation on free acid groups on the copolymer, thus reducing ester-based crosslinking (**Figure 3G**). The main difference that can be seen in the spectra of the crosslinked films in contrast with the non crosslinked ones, is the presence of a new band between 1800 and 1750 cm^−1^. This band can be attributed to the new ester bonds formed between the Gantrez S-97 acid groups and the hydroxyl groups of the PEG molecules. In addition a new band between 1500 and 1600 cm^−1^ can be observed for super-swelling hydrogels that is not present in the other hydrogels. This band is characteristic for the salts of carboxyilic acids [26].

To examine the controlling mechanism of swelling of the super swelling hydrogel materials prepared from aqueous blends of 20% w/w Gantrez S-97, 7.5% w/w PEG and 3% w/w Na_2_CO_3_, the second order kinetic model (**Equation 4**) was used to process the experimental data. To analyse the kinetic model, *t/S* versus *t* graphs were plotted and respective swelling rate parameters were determined. **Figure 3D** shows representative linear regression plots of the swelling curves derived from **Equation 4**. The diffusional exponent, n, was determined to be 0.76, indicating an anomalous mechanism of water uptake. In addition, the diffusion coefficient (*D_i_*) was 2.47×10^−6^ cm^2^ min^−1^. The volume fraction of polymer, 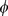, determined using **Equation 6** was 0.045, the number average molecular weight between crosslinks, M_c_, determined using **Equation 5**, was 6,793,627 g/mol. The crosslink density, *V_e_*, determined using **Equation 8** was 1.08×10^19^. Due to dissolution of other candidates, the formulation which was selected for continued investigation was that containing 20% w/w Gantrez S-97, 7.5% w/w PEG and 3% w/w Na_2_CO_3_.

### 3.2. Mechanical testing

MN arrays formulated using the super swelling formulation (20% w/w Gantrez S-97, 7.5% w/w PEG and 3% w/w Na_2_CO_3_) and in the geometry, 19×19 (height = 600 µm, width = 300 µm, interspacing = 50 µm) were used to investigate the effects of compression tests on the heights of individual needles on the MN array. The digital microscope images presented in **Figure 3E** are illustrative of the effects, on individual needles of the MN array, of the fracture forces applied by axial load. It is important to note that regardless of the force applied, none of the needles on the MN array broke or shattered upon application into the skin, rather bending. **Figure 3E** also shows the percentage reduction in the height of individual needles on the MN array upon application of increasing fracture forces. The reduction in MN height increased progressively with increases in the force applied. These MN deformed when applied to a stainless steel plate, but were not brittle, which is important from a patient safety point of view. This is especially true considering the relatively high forces applied here (>300 N was the maximal force applied over the 361 MN) and the much softer nature of skin.

**Figure 4:**
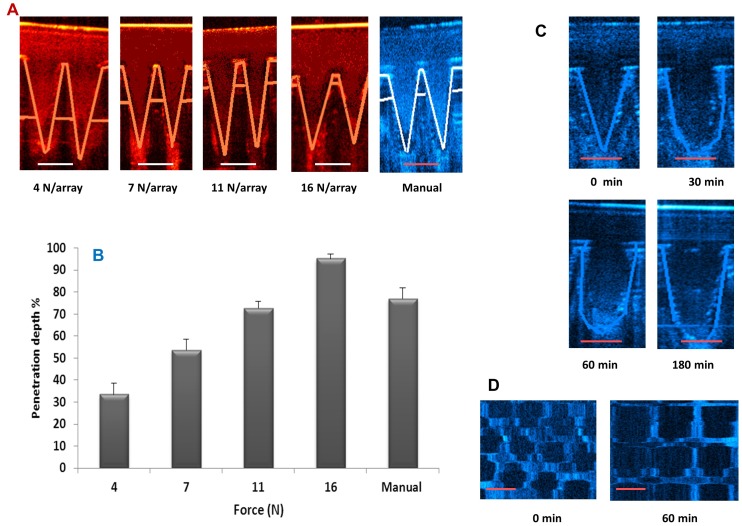
False colour 2D still images of super swelling MN arrays immediately following insertion into excised neonatal porcine skin at application forces of 4, 7, 11 or 16 N/array or using manual application. (Scale bar represents 300 µm in each case) (**A**). The effect of application force (N/array) upon the resultant penetration depth of super swelling MN arrays in neonatal porcine skin *in vitro*, expressed as a p [percentage of MN height (Means+S.D., n = 10)]. The penetration parameters of the MN arrays were quantified using optical coherence tomography (**B**). False colour images of the *in vitro* swelling profile of MN arrays in excised neonatal porcine skin recorded over a 3 h period, as assessed by optical coherence tomography (Scale bar represents 300 µm in each case) (**C**). OCT visualisation of the micropores residing within the skin immediately following MN array insertion (0 min) and following 60 min in skin (Scale bar represents 300 µm. in each case) (**D**).

Skin penetration of super swelling MN arrays was investigated using dermatomed neonatal porcine skin (approximately 350–450 µm thicknesses) and the percentage of holes (micro-conduits) created by the MN arrays was determined after staining of the skin with methylene blue solution. Regardless of force applied,>85% of the MN in each array penetrated the *stratum corneum*, as evidenced by staining of the formed aqueous microconduits by the hydrophilic marker compound. However, with increasing applied forces, the penetration efficiency of the MN also increased (**Figure 3F**). In the case of the 0.9 N/needle applied force, the microconduits created could be traced onto the surface of the laboratory film (Parafilm) placed beneath the skin. This indicated that, at this highest insertion force, which equates to 324.9 N/array as there are 361 needles in each array, the depth of penetration of the needles into the skin was at its greatest. In order to accurately measure miroconduit depth, optical coherence tomography was utilised. The penetration characteristics of MN arrays inserted into neonatal porcine skin using an applicator set to defined forces of 4, 7, 11 or 16 N/array are presented in **Table 2** and **Figure 4B**. Manual force (defined as “gentle finger pressure”) was also used to insert the MN arrays and the penetration characteristics are very similar to those quoted when a force of 11 N/array was employed. Increasing force increased penetration depth and decreased distance between the lower MN baseplate and the *stratum corneum*, but microconduit width was largely unaffected.

**Table 2:** The effect of force of application upon the resultant penetration characteristics of MN arrays cast from 20% w/w Gantrez S-97, 7.5% w/w PEG 10,000 and 3% w/w Na_2_CO_3_, in the geometry 19×19 with height 600 µm, width 300 µm and interspacing at base 50 µm into neonatal porcine skin, (Means ± SD, n = 10).

Force (N/array)	MN penetration depth (µm)	Pore width (µm)	Base plate/*Stratum corneum* distance (µm)
**4**	201±31	209±10	398±31
**7**	322±35	214±17	277±35
**11**	430±20	219±8	169±20
**16**	571±8	228±12	29±8
**Manual**	465±28	211±7	134±28

### 3.3. In skin swelling

The swelling of the MN arrays upon application into skin was then investigated *in vitro* over 3 hours and in real time. Individual needles on the arrays exhibited an increase in height of approximately 40% by the end of the three-hour testing period (**Figure 4C** and **Table 3**). The microconduits residing within the skin immediately and 60 min post-MN array application were visualised (**Figure 4D**). Importantly, these images confirm that skin under occlusion swells and relaxes with the MN, meaning their increase in volume does not result in the extravasation of the swollen MN from the skin.

**Table 3:** *In vitro* swelling of MN arrays (19×19 MN, 600 µm height, 300 µm width at base, 50 µm interspacing at base) upon insertion into neonatal porcine skin, (Means ± SD, n = 15).

Time (min)	MN depth in skin (µm)
0	465.25±28.25
30	578.08±22.74
60	609.50±35.13
180	697.27±41.63

### 3.4. Lyophilised drug reservoirs

Different OVA-loaded and ibuprofen sodium-loaded formulations were prepared and lyophilised. An OVA-loaded formulation containing 10% w/w gelatin (Sigma-Aldrich, Dorset, UK), 40% w/w mannitol (Sigma-Aldrich, Dorset, UK), 10% w/w NaCl, 1% w/w sucrose and 0.5% w/w OVA was determined to be the most suitable, in terms of morphology, strength and dissolution profile of the formed wafers and, hence, was chosen for further characterisation studies. In terms of the ibuprofen sodium, those wafers which were prepared from blends containing 10% w/w gelatin (Cryogel SG3, PB Gelatins, Pontypridd, UK), 3% w/w mannitol (Pearlitol 50C-Mannitol, Roquette, Lestrem, France) and 40% w/w ibuprofen sodium (Sigma-Aldrich, Dorset, UK) were chosen for subsequent investigation, as they were the most homogeneous. There was no loss of active in either case, with recoveries of 100% for ibuprofen sodium and 98±18% for OVA. In addition, the wafers all complied with pharmacopoeial standards [27] for hardness, weight variation, thickness and friability (**Table 4**).

**Table 4:** Physical properties of lyophilised drug reservoirs.

Parameter	Means ± S.D.
**Ovalbumin**	
Weight (g)	0.32±0.01
Hardness (N)	119.7±5.0
Thickness (mm)	4.12±0.3
Friability	0.47% mass loss
**Ibuprofen sodium**	
Weight (g)	0.26±0.02
Hardness (N)	178±3
Thickness (mm)	4.96±0.6
Friability	0% mass loss

### 3.5. *In vitro* drug release studies

**Figure 5:**
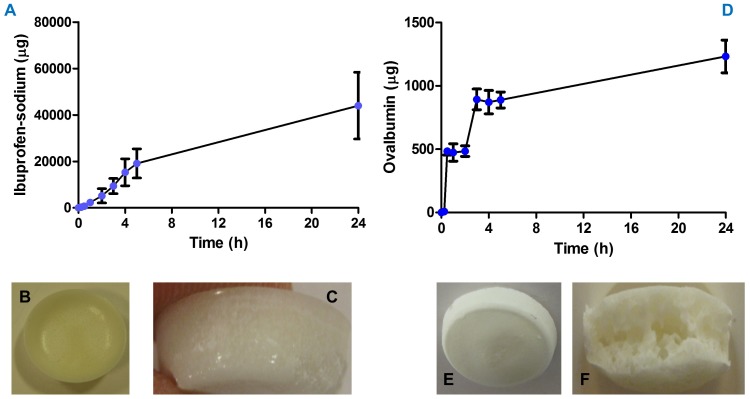
The *in vitro* cumulative permeation profile of ibuprofen sodium across dermatomed 350 µm neonatal porcine skin when delivered using in-dwelling super swelling MN arrays combined with lyophilised drug reservoirs (Means ± S.D., n = 9) (A). Digital images of the ibuprofen sodium-loaded lyophilised wafers used in *in vitro* and *in vivo* experiments and prepared from aqueous blends containing 10% w/w gelatin, 3% w/w mannitol and 40% w/w ibuprofen sodium (**B**, **C**). The *in vitro* cumulative permeation profile of OVA across dermatomed 350 µm neonatal porcine skin when delivered using in-dwelling super swelling MN arrays combined with lyophilised drug reservoirs (Means ± S.D., n = 5) (**D**). Digital images (**E**, **F**)of the OVA-loaded lyophilised wafers used in *in vitro* and *in vivo* experiments and prepared from aqueous blends containing 10% w/w gelatin, 40% w/w mannitol, 10% w/w NaCl, 1% w/w sucrose and 0.5% w/w OVA. These active-loaded tablets exhibited high porosities as exemplified in (**G**).

Ibuprofen sodium exhibited an almost typical first order release profile across excised neonatal porcine skin *in vitro* (**Figure 5A**), with approximately 44 mg delivered in 24 h (range from 9 replicates: 35.2–68.9 mg over 24 h). As the ibuprofen sodium-loaded reservoirs were known to contain a mean loading of 124 mg approximately 37% of this was delivered in 24 h. OVA exhibited a tri-phasic release profile (**Figure 5D**), possibly due to the very different composition and morphology of the lyophilised wafers (**Figure 5F**) and the much greater molecular weight and more complex molecular structure, as compared to ibuprofen sodium. The average total OVA content of the lyophilised wafers was 2.5±0.15 mg and it was found that the super swelling MN arrays delivered approximately 1.24 mg OVA over the 24 h experimental period (range from 5 replicates: 1.09–1.36 mg over 24 h). This equates to transdermal delivery of approximately 49% of the OVA loaded into the wafers on average. This is interesting to note, since such effective *in vitro* transdermal delivery has not been seen previously for either high dose low potency molecules or proteins. However, it is unsurprising, given the high molecular weight between crosslinks of the super swelling hydrogel calculated at equilibrium (6,793,627 g/mol) compared to the molecular weights of ibuprofen sodium (229.29 g/mol) and even ovalbumin (44,300 g/mol). Drug permeation is thus likely to be affected more by the dissolution rate of the lyophilised wafers than the hydrogel material, assuming in-skin swelling is complete.

### 3.6. *In vivo* experiments

**Figure 6:**
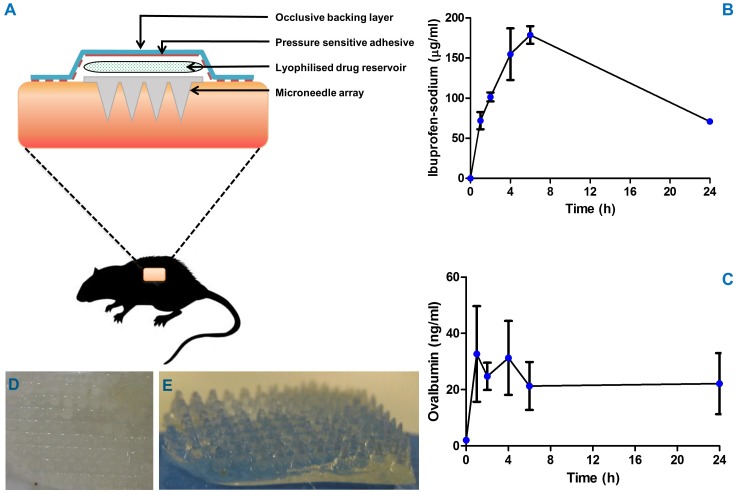
Schematic representation of application and retention strategies for rat experiments designed to evaluate *in vivo* performance of super swelling microneedle arrays (A). The *in vivo* plasma profiles of ibuprofen sodium (**B**) (Means ± S.D., n = 4) and OVA (**C**) (Means ± S.D., n = 3) following transdermal delivery using super swelling microneedle arrays with lyophilised drug reservoirs. Typical morphology of super swelling microneedles upon removal from rat skin *in vivo* after 24 hours insertion indicating that, despite extensive swelling, the microneedles are removed intact (**D**, **E**).

In the case of experiments carried out using OVA-loaded reservoirs, two super swelling MN arrays and active-loaded wafers were applied to the backs of the animals (**Figure 6A**). In contrast, four MN arrays and their respective ibuprofen sodium-loaded reservoirs were applied to the backs of the animals in parallel drug delivery experiments. Plasma profiles in the case of both model compounds differed from the patterns of drug permeation profiles seen *in vitro*. This is unsurprising, given that the two experiments are distinct from one another, since the *in vitro* experiments do not have biodistribution, metabolism or excretion components as we have *in vivo*.

For ibuprofen sodium (**Figure 6B**), the integrated MN array delivery system produced a progressive increase in plasma concentrations over 6 h, with a maximal concentration of approximately 179 µg/ml achieved in this time. The plasma concentration had fallen to 71+6.7 µg/ml by 24 h. Therapeutic plasma levels of ibuprofen in humans range between 10 and 15 µg/ml [28] and these levels were achieved within the first hour of MN application. Based on this knowledge and the *in vivo* results, we can approximate the patch size necessary for use in human volunteer studies. An average human male weighs approximately 60 kg [29], which is 286 times greater than the weight of a 210 g rat (the average weight of rat used in these experiments). The peak plasma ibuprofen sodium concentration achieved in the rats at 6 h (179±19 µg/ml) is approximately 18 times greater than the human therapeutic blood levels [28] and this was achieved with MN arrays of total approximate area of 2 cm^2^ (4×0.5 cm^2^). By this rationale, a MN patch design of no greater than 32 cm^2^ could potentially deliver therapeutically-relevant doses of ibuprofen sodium in healthy volunteer studies. Typical commercialised transdermal patches can be as large as 30 or 40 cm^2^ (Novartis make Nicotinell nicotine patches of 30 cm^2^ [30]; Janssen make Duragesic CII (fentanyl) patches of 32 and 42 cm^2^ [31]). Accordingly, it is very reasonable to suggest that a MN product could be successfully developed based on the technology and data presented here. Indeed, we have previously shown that scaling up MN patch size is a relatively straightforward process [8], [10].

OVA levels peaked in plasma after only 1 hour (**Figure 6C**) at 42.36±17.01 ng/ml. This represents a significant finding, since macromolecules are normally absorbed quite slowly when administered intradermally [4], [5] OVA plasma levels then remained almost constant up to 6 h, dropping somewhat at 24 h, when 23.61±4.84 ng/ml was detected.

Importantly, the super swelling hydrogel MN arrays remained intact over the 24 h application period in all cases, thus allowing their removal as an intact unit at the end of the experiment (**Figure 6D, E**). As can be appreciated, the MN array extensively absorbed interstitial fluid to form a swollen hydrogel matrix, thus enabling delivery of OVA and ibuprofen sodium across the skin and into the systemic circulation. However, the system was sufficiently robust when swollen to ensure that the MN were removed intact.

## Conclusions

The work presented here shows, for the first time, that exploitation of so-called “super swelling” hydrogel materials in microneedle-enhanced transdermal drug delivery is a highly promising approach to increasing the range of drugs that can be delivered transdermally. Using such systems in combination with lyophilised wafer-type drug reservoirs facilitated delivery of doses of ibuprofen sodium that could be extrapolated to a useful and usable product for human treatment. Rapid protein delivery using ovalbumin as the model indicated that this technology may also find use in macromolecular drug delivery and vaccine administration. The value to industry is likely to be considerable, since this technology is distinctly different from conventional microneedle systems, which are presently only suitable for bolus delivery of very potent drugs and vaccines. Accordingly, we are currently progressing towards clinical evaluations with a range of candidate molecules.
